# Age-Associated Dysregulation of Integrin Function in Vascular Smooth Muscle

**DOI:** 10.3389/fphys.2022.913673

**Published:** 2022-07-07

**Authors:** Krishna Raj Ojha, Song Yi Shin, Samuel Padgham, Frida Leon Olmedo, Bohong Guo, Gang Han, Christopher Woodman, Andreea Trache

**Affiliations:** ^1^ Department of Medical Physiology, Texas A&M University Health Science Center, Bryan, TX, United States; ^2^ Department of Health and Kinesiology, Texas A&M University, College Station, TX, United States; ^3^ Department of Biomedical Engineering, Texas A&M University, College Station, TX, United States; ^4^ Department of Epidemiology and Statistics, Texas A&M University Health Science Center, College Station, TX, United States

**Keywords:** aging, integrins, actin, vascular smooth muscle, atomic force microscopy

## Abstract

Arterial aging results in a progressive reduction in elasticity of the vessel wall and an impaired ability of aged blood vessels to control local blood flow and pressure. Recently, a new concept has emerged that the stiffness and decreased contractility of vascular smooth muscle (VSM) cells are important contributors to age-induced arterial dysfunction. This study investigated the hypothesis that aging alters integrin function in a matrix stiffness-dependent manner, which contributes to decreased VSM contractility in aged soleus muscle feed arteries (SFA). The effect of RGD-binding integrins on contractile function of cannulated SFA isolated from young (4 months) and old (24 months) Fischer 344 rats was assessed by measuring constrictor responses to norepinephrine, phenylephrine, and angiotensin II. Results indicated that constrictor responses in presence of RGD were impaired in old compared to young SFA. VSM cells isolated from young and old SFA were used for functional experiments using atomic force microscopy and high-resolution imaging. Aging was associated with a modulation of integrin β1 recruitment at cell-matrix adhesions that was matrix and substrate stiffness dependent. Our data showed that substrate stiffening drives altered integrin β1 expression in aging, while soft substrates abolish age-induced differences in overall integrin β1 expression. In addition, substrate stiffness and matrix composition contribute to the modulation of SMα-actin cytoskeleton architecture with soft substrates reducing age effects. Our results provide new insights into age-induced structural changes at VSM cell level that translates to decreased functionality of aged resistance soleus feed arteries.

## 1 Introduction

Preventing age-induced arterial dysfunction and the associated risk of cardiovascular disease remains a significant clinical challenge (Lakatta, 2013). Aging is an independent risk factor for cardiovascular disease with high incidence in patients 65 and older (Virani et al., 2020). Arterial stiffening in aging leads to decreased vascular smooth muscle (VSM) cell contractility and induces hyperplastic remodeling of aged arteries (Rizzoni et al., 2000; Najjar et al., 2005; Briones et al., 2007).

In aging, an increase in pulse pressure that is transmitted from large conduit arteries to small resistance vessels followed by an overall increase in pressure in small arteries can lead to organ damage (O'Rourke and Safar, 2005). Indeed, clinical studies have shown that arterial stiffness is directly related to aging (Mitchell et al., 2004; AlGhatrif et al., 2013) and hypertension (Tomiyama et al., 2004). While age-induced vessel stiffening studies are mostly directed towards conduit vessels, resistance vessels also become stiffer with age (Gothberg and Folkow, 1983). Recent evidence indicates changes in the extracellular matrix alone are not sufficient to fully account for increased vascular stiffness and loss of contractility in aged arteries and suggest that intrinsic mechanical properties of VSM cells are important contributors to arterial stiffening and vasomotor dysfunction in aging and cardiovascular disease. An emerging concept indicates that stiffening of VSM cells contributes to decreased contractility, a hallmark of age-induced arterial dysfunction (Sehgel et al., 2015a; Zhu et al., 2018).

VSM cells regulate vessel wall contractility through interactions between the actomyosin and integrin-based cell-matrix adhesions that anchor the cells within the surrounding extracellular matrix (Lakatta, 2013; Saphirstein et al., 2013). Integrin activation can be initiated by forces transmitted through matrix stiffening (outside-in signaling) or by changes in the cytoskeletal tension (inside-out signaling) (Ginsberg et al., 1992; Hynes 1992; Hynes 2002). The integrin subunits most prevalent in VSM cells include α1, α2, α5 and αv, as well as β1, β3 and β5 (Moiseeva, 2001). The formation of functional integrin dimers define the affinity of the receptor for specific extracellular matrix proteins, for example, α5β1 and αvβ3 recognize the RGD sequence on fibronectin, while α1β1 and α2β1 recognize the GFOGER sequence on collagens (Ruoslahti 1996; Zeltz and Gullberg 2016). In turn, extracellular matrix composition induces expression of specific integrins, such that signaling pathways are activated in a controlled manner and further induce specific cell responses (Seetharaman and Etienne-Manneville, 2018). Thus, integrin α5β1, the main fibronectin receptor, is involved in the regulation of contractile function in VSM cells (Martinez-Lemus et al., 2003; Martinez-Lemus et al., 2005a) because its activation mediates force generation by increasing myosin II activity via RhoA/ROCK pathway (Schiller et al., 2013). Integrin αvβ3 also has a role in cell adhesion to fibronectin with a lower affinity than α5β1. Since integrin αvβ3 and α5β1 are spatially separated at the basal cell membrane in the presence of fibronectin, it has been proposed that activity of this integrin is regulated by force-dependent cell morphology (Felsenfeld et al., 1999). Moreover, integrins α5β1 and αvβ3 have distinct roles in regulating vessel wall contractile function (Martinez-Lemus et al., 2003; Martinez-Lemus et al., 2005b; Martinez-Lemus et al., 2009). Integrin α5β1 activation by RGD binding plays a role in regulating arterial smooth muscle contraction (Wu et al., 1998; Wu et al., 2001), while integrin αvβ3 binding to the same ligand regulates arterial smooth muscle relaxation (D’Angelo et al., 1997). However, both integrins are activated during vasoconstrictor responses to increased pressure (Martinez-Lemus et al., 2005b).

Local changes in matrix microenvironment trigger mechanical signaling to VSM cells which in turn is transformed into a physiological response termed mechanotransduction. The age-induced environmental mechanical cues can drive the phenotype switch for VSM cells, from a contractile to a synthetic phenotype characteristic of aging. In addition, cell-matrix adhesions and cytoskeleton tension are considered main players in the mechanotransduction process (Discher et al., 2005). Recently, VSM cell stiffness and adhesion properties have been recognized as important contributors to the arterial stiffness in aging (Sehgel et al., 2015a; Lacolley et al., 2017). Thus, integrin clustering at cell-matrix adhesions is directly dependent of matrix stiffness. Substrate mechanical properties provide key physical cues for the regulation of cell adhesions and further intracellular signaling to enable cell adaptation to its microenvironment (Fusco et al., 2015). A stiffer microenvironment characteristic of aging is sensed as a high mechanical tension element by the integrins which will favor integrin clustering and organization of adhesion patterns (Gupta et al., 2015). More recently, actin cytoskeleton remodeling was recognized as another important factor contributing to the cellular adaptive response. Formation of filamentous actin further contributes to cellular stiffening, hence, high cytoskeletal tension. In contrast, on softer substrates integrins sense a lower mechanical tension which favors only limited integrin clustering and differential actin organization throughout the cell (Gupta et al., 2015; Gupta et al., 2016). Since vascular aging presents a high level of heterogeneity throughout the arterial network (Barton et al., 1997; Kohn et al., 2016; Trache et al., 2020), understanding the correlation between local, passive tissue properties (i.e., matrix stiffness) and active components of the vascular wall (i.e., cell stiffness) is of high intertest in elucidating the age-induced arterial stiffening and vessel wall remodeling. Both collagen-I and fibronectin are increased in aging (Lakatta, 2013). An increase in fibronectin is thought to be related with an increase in cell adhesion (Qiu et al., 2010), while an increase in collagen-I may be a potential trigger for changes in the VSM cell phenotype due to alterations of mechanical properties of the matrix in the vessel wall (McDaniel et al., 2007).

In the present study we hypothesized that aging alters integrin function in a matrix stiffness-dependent manner, which contributes to decreased VSM contractility in aging. Thus, we combined high-resolution fluorescence imaging of VSM cell structural changes with functional approaches both at cell and vessel level. Our results provide new insights into age-induced structural changes at VSM cell level that translate to decreased functionality of aged resistance arteries.

## 2 Materials and Methods

### 2.1 Animals

Experimental protocols were approved by the Texas A&M University Institutional Animal Care and Use Committee. Male Fischer 344 rats, both young (4 months) and old (24 months), were obtained from the National Institute on Aging (NIA). Animals were housed at the Texas A&M comparative medicine program facility and kept under a 12:12 h light-dark cycle with free access to food and water. Research staff and Animal Care Facility veterinarians supervised the health of the animals 7 days a week.

### 2.2 Isolation and Cannulation of Soleus Muscle Feed Arteries

#### 2.2.1 Isolation of Soleus Muscle Feed Arteries

Soleus muscle feed arteries (SFA) were isolated as described previously (Seawright et al., 2018). In brief, rats were sedated with ketamine (80 mg/kg body weight) and Xylazine (5 mg/kg body weight) using an intraperitoneal injection. Anesthesia was verified by an unresponsive toe-pinch. The soleus-gastrocnemius-plantaris muscle complex was removed from each hindlimb and placed in cold (4°C) MOPS-buffered physiological saline solution (PSS) containing: 145 mM NaCl, 4.7 mM KCl, 2 mM pyruvate, 2 mM CaCl_2_, 1.17 mM MgSO_4_, 1.2 mM NaH_2_PO_4_, 5 mM glucose, 2 mM pyruvate, 0.02 mM EDTA, and 25 mM MOPS (pH 7.4). SFA were isolated, dissected, and placed in a Lucite chamber containing MOPS-PSS (pH 7.4 at 4°C) for cannulation. Rats were euthanized by excising the heart.

#### 2.2.2 Cannulation of Soleus Muscle Feed Arteries

SFA were cannulated on each end with a glass micropipette and secured with surgical thread (Seawright et al., 2016). Each micropipette was attached to a pressure reservoir filled with MOPS-PSS supplemented with albumin (1 g/100 ml). SFA were initially pressurized to 60 cmH_2_O (1 mmHg = 1.36 cmH_2_O) for 20 min and checked for leaks by verifying that intraluminal diameter remained constant after closing the pressure reservoirs. Once the vessel was determined to be leak-free, pressure in the SFA was raised to 90 cmH_2_O for an additional 40 min. PSS was replaced at 20-min intervals and temperature of the vessel chamber was maintained at 37°C throughout the experiment. To determine maximal passive diameter, SFA were incubated in Ca^2+^ free PSS for a minimum of 30 min.

### 2.3 Assessment of Soleus Feed Artery Constrictor Responses to RGD Integrin Blockade

Vasoconstrictor responses of SFA were assessed by measuring the change in diameter in response to cumulative, increasing, whole log additions of norepinephrine (NE), phenylephrine (PE) and angiotensin II (Ang II) (Seawright et al., 2018). NE was assessed at concentrations ranging from (10^–9^ x 10^–4^ M) and was used to stimulate α-1 and α-2 adrenergic receptors, PE (10^–9^ x 10^–4^ M) selectively stimulated α-1 adrenergic receptors, and Ang II (10^–11^ x 10^–7^ M) stimulated angiotensin (AT) receptors. NE and PE were obtained from Sigma-Aldrich (St. Louis, MO) and Ang II was acquired from Bachem (Torrance, CA).

To evaluate the contribution of integrins to constrictor function, responses to NE, PE, and Ang II were assessed in the absence or presence of a functional integrin blocking cyclo-(GRGDSP) peptide or a cyclo-(GRGESP) as control peptide (Anaspec, Fremont, CA), both used at 210 μM. Each peptide was added to the vessel bath, and incubated for 20 min.

Two-Way ANOVA was used to determine whether constrictor responses to NE, PE, or Ang II differed by group. Data are presented as percent constriction and calculated as [(D_b_-D_c_)/D_b_] X 100, where D_c_ is the measured diameter following administration of an agonist. D_b_ is the baseline diameter prior to administering the agonist. When a significant *p*-value was obtained, post hoc analyses were performed with Duncan’s Multiple-Range Test. All values are presented as mean ± SE. Statistical significance was defined as *p* < 0.05.

### 2.4 Vascular Smooth Muscle Cell Culture

VSM cells were isolated from SFA obtained from young and old Fischer 344 rats as previously described (Seawright et al., 2018). Low passage cells were cultured in 5% CO_2_ at 37°C in Dulbecco’s Modified Eagle Medium F-12 supplemented with 10% fetal bovine serum and 10 mM HEPES (Sigma, St. Louis, MO), 2 mM L-glutamine, 1 mM sodium pyruvate, 100 U/ml penicillin, 100 μg/ml streptomycin and 0.25 μg/ml amphotericin B. All reagents were purchased from Invitrogen (Carlsbad, CA), unless otherwise specified. Cells were plated on glass bottom dishes (MatTek, Ashland, MA) functionalized with 20 μg/ml matrix proteins (collagen I (Coll I) or fibronectin (FN)). Uncoated dishes were used as control (Lim et al., 2010). All extracellular matrix proteins used for cell culture were purchased from Sigma-Aldridge (Saint Louis, MO).

To further determine substrate stiffness effects on aged VSM cells, silicone sheeting (SMI, Saginaw, MI) was cut to fit a custom-made acrylic frame as previously described (Na et al., 2008). Silicone membrane, tools, and frame were sterilized in an ethanol bath followed by UV exposure for 20 min. After the frames were sterilized and dried, autoclaved vacuum grease was brushed on to the frame. The membrane was laid flat, and the greased portion of the frame was placed over the membrane. The membrane assembly was inverted and fixed in place with a locking ring. Once assembled, the membranes were coated with matrix proteins (FN or Coll-I) and incubated for 3 h at 37°C.

### 2.5 Real-Time Quantitative Polymerase Chain Reaction

VSM cells isolated from soleus feed arteries of young and old Fischer 344 rats were subjected to total RNA extraction using TRIzol™ (Invitrogen, Carlsbad, CA) following the manufacturer’s protocol. Briefly, cells were grown to confluency on 60 mm cell culture dishes yielding 500,000 cells/dish. The media was removed and 1 ml of TRIzol™ was added directly to the culture dish to lyse the cells. The mixture was homogenized by pipetting and the lysates were transferred to 1.5 ml Eppendorf tubes for isolation. Lysates were mixed with chloroform and centrifuged at 12,000 × g to separate the sample into the phenol-chloroform phase, interphase, and the aqueous phase. RNA was precipitated from the aqueous phase with the addition of isopropyl alcohol and pelleted via centrifugation. RNA pellets were washed twice with 75% ethanol and allowed to air dry. Pellets were resuspended in nuclease-free water and RNA samples were quantified via Qubit RNA broad range assay (Invitrogen, Carlsbad, CA) and RNA quality was assessed via Agilent TapeStation RNA Screentape (Agilent, Santa Clara, CA).

RNA samples were normalized to the same concentration and approximately 400 ng of RNA was input into the RT^2^ First Strand Kit (Qiagen, Germantown, MD) following the manufacturer’s recommended protocol for downstream use in the RT^2^ Profiler PCR Array format E 384 (4 × 96). Gene expression was assessed with the RT^2^ Profiler PCR Array for Rat Focal Adhesions (Qiagen PARN-145ZE-1, Germantown, MD) and measured on a Bio-Rad CFX384 real time PCR instrument (Bio-Rad, Hercules, CA). The real-time PCR thermal program comprised polymerase activation at 95°C for 10 min, 40 cycles of denaturation at 95°C for 15 s, annealing, and extension at 60°C for 60 s. A recommended melting curve analysis was also performed to verify PCR specificity identifying a single peak in each reaction at temperatures greater than 80°C. The parameters for melt curve analysis were 95°C for 1 min, 65°C for 2 min, then, the temperature was increased at a rate of 2°C/min from 65°C to 95°C. The resulting C_t_ values were imported into Qiagen’s GeneGlobe Data Analysis Center and assessed for differential gene expression. The comparative threshold (C_t_) method (2^-∆∆Ct^) was employed to evaluate relative expression levels (Livak and Schmittgen, 2001). Data are shown as mean ± SEM with *p* < 0.05 being considered statistically significant.

Smooth muscle α-actin (*Acta2*) was assessed separately. Approximately 600 ng RNA was reversed transcribed with SuperScript II reagents (ThermoFisher Scientific, Waltham, MA) and ReadyMade oligo dT_(20)_ primer (Integrated DNA Technologies, Coralville, IA) at 2.0 µM final concentration, and the cDNA was diluted 1:4 in nuclease-free water. PCR reactions were performed in triplicate with 20 µl reaction volume containing 2 µl cDNA, 10 µl of 2X TaqMan^®^ Universal PCR Master Mix (no uracil-N-glycosylase; ThermoFisher Scientific, Waltham, MA) and 1 µl of the target gene TaqMan^®^ qPCR assay (ThermoFisher Scientific, Waltham, MA). Same thermal cycling conditions were used as described above. Ubiquitously expressed prefoldin-like chaperone (*Uxt*) was utilized as the reference gene for calculating relative expression. TaqMan^®^ assay IDs are Rn01759928_g1 (*Acta2*) and Rn01430624_m1 (*Uxt*). Relative expression was calculated by ΔΔCt method as described above.

### 2.6 Adhesion Assays

Polystyrene micro-plates with 96-wells (Corning-Costar, Cambridge, MA) were functionalized with 20 μg/ml fibronectin (Sigma, St. Louis, MO) and incubated at 4°C overnight followed by blocking for 30 min at room temperature with 1% bovine serum albumin (Sigma-Aldridge, St. Louis, MO) as previously described (Lim et al., 2010). Briefly, cells were incubated in suspension with RGD or RGE peptides (Anaspec, Fremont, CA) at a concentration of 0.8 mM for 15 min at 37°C in serum free media. Cells without any peptide treatment plated on uncoated substrates blocked with BSA were used as controls. Experiments were performed in triplicate with 30,000 cells added to each well and incubated at 37°C and 5% CO_2_. Cells were allowed to attach to the substrate for 20 min, and unattached cells were removed by washing the plate with DPBS supplemented with 1 mM Ca^2+^ and 1 mM Mg^2+^. Fixed cells were stained with 0.1% Amido black (Sigma-Aldridge, St. Louis, MO), and then cells were rinsed in distilled water and the dye was eluted with 2 N NaOH. The optical density for each microwell was quantified at 595 nm using a Synergy HT-1 plate reader (Bio-TEK, Winooski, VT).

### 2.7 Assessment of VSM Cell Morphology

#### 2.7.1 Immunofluorescence Staining

VSM cells were plated on 35 mm glass bottom dishes or silicone membrane assemblies functionalized with matrix proteins as described above. Dishes were washed with Dulbecco’s Phosphate buffered saline (DPBS) before plating the cells. After 24 h, cells were fixed for 10 min in 2% paraformaldehyde (Electron Microscopy Sciences, Hatfield, PA), in DPBS followed by washing with glycine buffer. After fixation, cells were treated with protein specific antibodies in a sodium citrate buffer containing 1% w/v BSA and 0.1% v/v Triton-X and incubated at 4°C overnight (Sun et al., 2005). Then, cells were washed in a sodium citrate buffer with 5 min incubation at room temperature and further incubated with secondary antibodies for 1 h at room temperature (Seawright et al., 2018). After this time, cells were again thoroughly washed, immersed in DPBS, and then imaged. The following antibodies were used: rabbit anti-integrin α2 (Abcam, Boston, MA); rabbit anti-integrin α5 (Millipore, Temecula, CA); hamster anti-integrin-β1 conjugated with Alexa 488 and hamster anti-integrin-β3 conjugated with Alexa 488 (Biolegends, San Diego, CA); mouse anti-smooth muscle α-actin (Sigma, St. Louis, MO, United States); goat anti-mouse Alexa 568; goat anti-rabbit Alexa 488 or Alexa 568 (ThermoFischer Scientific, Waltham, MA).

#### 2.7.2 VSM Cell Imaging

Total internal reflection fluorescence (TIRF) imaging of the cells was carried out on the home-built integrated microscope system described in (Trache and Lim, 2009). Briefly, an Olympus IX81 microscope (Olympus, Tokyo, Japan) equipped with a PLAN APO 60X oil 1.45 NA objective lens and fitted with a fiber optic laser launcher and a CoolSnap HQ CCD from Teledyne Photometrics (Tucson, AZ) was used for TIRF imaging. Thus, TIRF imaging allows visualization of cell-matrix adhesions in the immediate proximity of cell-substrate interface by providing super-resolution imaging in *z*-axis only (i.e., 100 nm) (Trache and Meininger, 2008). TIRF imaging was used to visualize the fluorescent-labeled integrins at cell-matrix interface for cells plated on glass bottom cell culture dishes with an exposure time of 100 ms.

Confocal imaging allows visualization of the whole cell body throughout the cytoplasm, including cell-matrix adhesion area. Thus, confocal imaging was used to image VSM cells stained for different integrins as well as actin. An Olympus Fluoview FV3000 Confocal Microscope equipped with a UPLSAPO 40XS silicon oil 1.25 NA objective lens was used in sequential mode scanning for dual color imaging of the whole cell. 3D confocal images were acquired as stacks of 12–14 planes at a 0.25 μm step size with an exposure time of 100 ms and are presented as xy projections. Same experimental parameters were used to acquire fluorescence images for each condition.

#### 2.7.3 Fluorescence Image Analysis

Slidebook software version 6 (Intelligent Imaging Innovations, Denver, CO) was used to quantify specific protein area as previously described (Lim et al., 2010). Measurements of the protein area at cell-matrix adhesions were performed on TIRF images, while total fluorescence intensity measurements throughout the cell were obtained from projections of confocal images. MatLab software version R2019a (Natick, MA) was used to determine the area for either the inner or peripheral region of each cell. To measure peripheral vs. inner protein area from fluorescence images, a mask was created that covered the entire interior of the cell with the cell membrane as boundary. Then, the mask was reduced in size to 75%, and positioned so that the centroid of the reduced mask coincided exactly with that of the original mask. Application of the reduced mask to the original image resulted in a new image that retained only the inner region of the cell for further processing. Alternatively, convolution of the original mask with the image complement of the reduced mask resulted in a new mask that, when applied to the original image, removed the interior portion of the cell, leaving only the peripheral region available for measurements.

#### 2.7.4 Statistical Analysis

Imaging experiments used VSM cells isolated from SFA from Fischer 344 rats (*n* = 2-4 animals) for each experimental condition. To statistically compare a large number of cells, fluorescence protein area was normalized to total cell area for each cell before statistical analysis by student’s t-test and ANOVA. Prior to running ANOVA, the normality assumption was checked using Q-Q plot and Shapiro-Wilk test. Linear regression was used to evaluate the interaction between age and substrate stiffness for each extracellular matrix. Estimated model coefficients with 95% confidence intervals were reported. Statistical significance was claimed if a significance level alpha was less than or equal to 0.05. SAS software version 9.4 (SAS Inc., Cary, NC) and STATA software version 16.0 (Stata LLC, College Station, TX) were used in this analysis.

### 2.8 Atomic Force Microscopy Measurements

Atomic force microscopy (AFM) measurements were performed on live VSM cells submerged in cell culture medium. The MLCT-Bio probes (Bruker Nano Surfaces Inc., Santa Barbara, CA) with a spring constant of 12.2 ± 0.4 pN/nm were used. The probe was washed with acetone followed by distilled deionized (DDI) water, and then coated with 10 mg/ml of PEG to further cross-link the fibronectin (1 mg/ml, ThermoFisher Scientific, Waltham, MA) onto the probe. The probes were thoroughly washed with DDI water after each step of the functionalization procedure as previously detailed in (Trache and Meininger, 2008). The probe was set to approach and retract from the cell surface at 0.8 µm/s. Cell culture medium containing RGD or RGE peptides (Anaspec, Fremont, CA) at a concentration of 0.8 mM were added to the cell culture dish and incubated for 45 min at room temperature. Experiments were performed in duplicates, and cells were randomly selected from the cell culture dish for a total of 750–1,500 individual force curve measurements for each experimental condition. The adhesion force was calculated using NForceR software (Trzeciakowski and Meininger, 2004) by multiplying the change in deflection height associated with the unbinding event by the spring constant of the cantilever. Then, PeakFit software version 4.11 (Systat Software Inc.) was used to estimate the associated confidence intervals for each distribution. Peaks whose confidence intervals did not overlap were considered significantly different (*p* < 0.05) (Ripley and Venables, 1994). The number of adhesion and non-adhesion events were counted, and the number of adhesion events was expressed as percent of total adhesions (Trache et al., 2005).

## 3 Results

### 3.1 Characteristics of Rats and SFAs

Young rats (357 ± 45 g) weighed significantly less than old rats (400 ± 45 g). The maximal passive diameters of SFA were not statistically different between young (186 ± 100 μm) and old (182 ± 70 μm) SFA.

### 3.2 Integrin-Mediated Vasoconstrictor Responses are Impaired With Aging

To determine the RGD-integrin binding contribution to SFA contractile function in aging, constrictor responses were assessed in the presence of RGD inhibitory peptide or RGE control, a non-inhibitory peptide. Two parallel studies were designed such that in one experiment, SFA constrictor responses to NE, PE, and Ang II were assessed in the presence of RGD inhibitory peptide, while in the second experiment, same constrictor responses were assessed in the presence of the RGE control peptide. Each of the peptides was added to the vessel bath at 210 μM for 20 min prior to assessing contractility responses. Moreover, another control experiment was designed to determine the effect of aging on constrictor function in SFA, by exposing arteries to the same cumulative increase of different contractile agonists (NE, PE, and Ang II) in the absence of any peptide.

**FIGURE 1:**
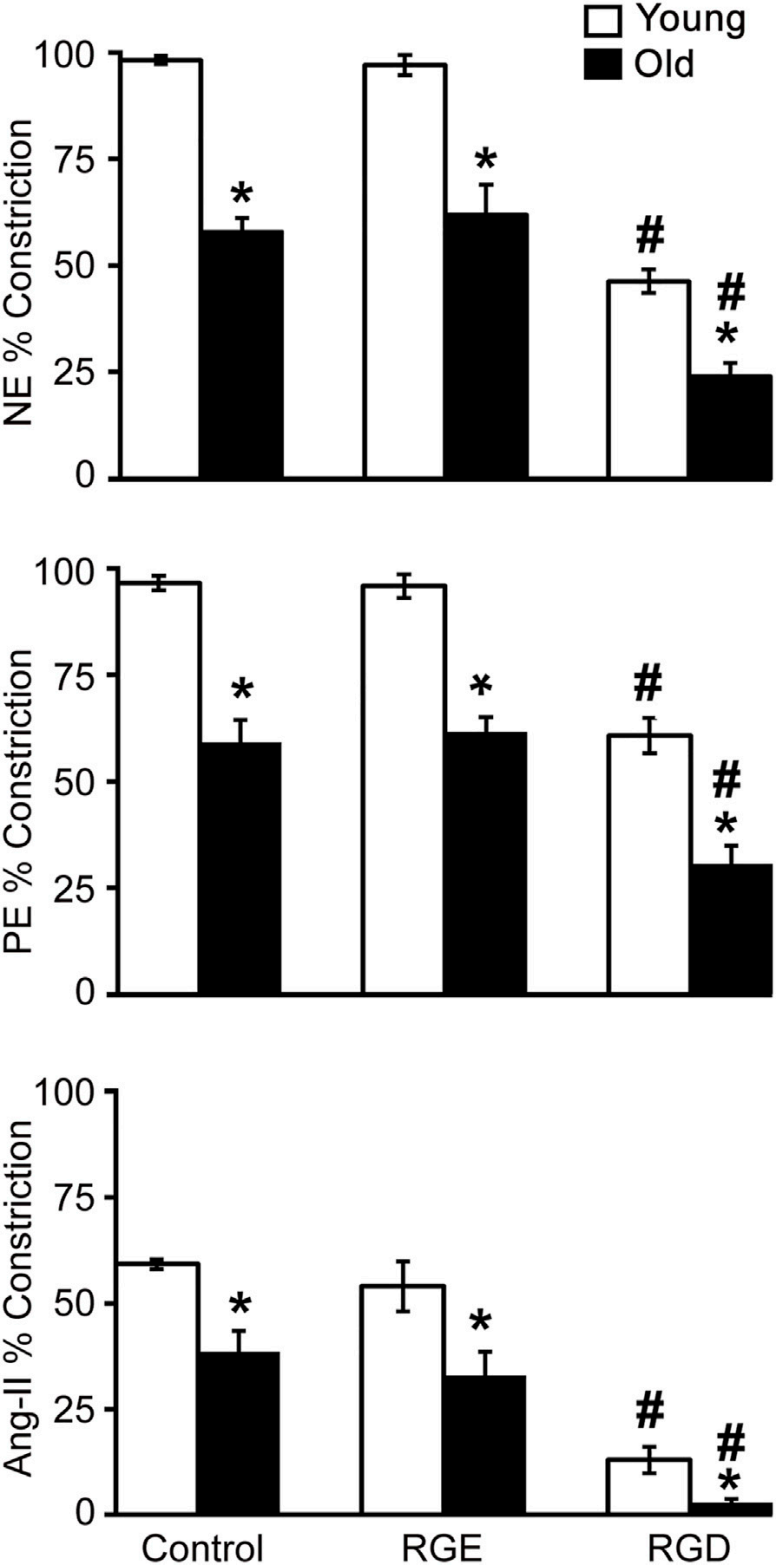
The role of integrin signaling in agonist-induced constrictor responses with age was assessed in the presence of a functional integrin blocking RGD peptide, a non-blocking RGE peptide, and control no treatment condition. Maximal constrictor responses to NE (10^–4^ M), PE (10^–4^ M), and Ang II (10^–7^ M) in SFA are presented. *n* = 5–12 rats per group. Data shown are mean ± SE. Significance was evaluated at *p* < 0.05. *Values are significantly different from young. # Values are significantly different from age-matched control and RGE group.

Figure 1 shows that vasoconstrictor responses to NE, PE and Ang II were less in old control SFA compared to young control SFA. Pre-treatment of SFA with RGE, a non-inhibitory peptide, did not alter constrictor responses to any of the agonists. Pre-treatment of SFA with RGD, an inhibitory peptide, reduced vasoconstrictor responses to all three agonists in young and old SFA. The finding that NE and PE-induced constriction was attenuated (not abolished) in the presence of RGD indicates that integrin signaling contributes to, but does not fully account for, adrenergic receptor-mediated constrictor responses in SFA (Supplementary Table S1). Results indicating that the RGD peptide nearly abolished constrictor responses to Ang II suggests that angiotensin receptor-mediated dilation is mostly dependent on integrin signaling in old SFA.

### 3.3 Aging Modulates Integrin Functional Properties in VSM Cells

#### 3.3.1 Aging Alters Integrin Expression and Recruitment at Cell-Matrix Adhesions

**FIGURE 2:**
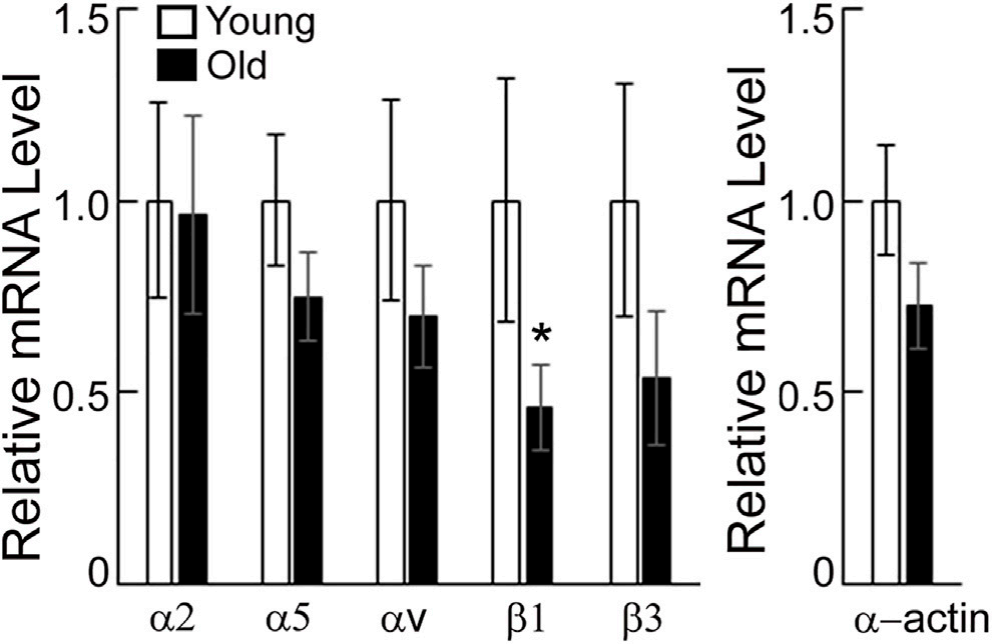
PCR assays were performed on VSM cells isolated from young and old SFA. Relative mRNA expression levels from young and old VSM cells were expressed relative to young VSM cells for each gene. Data are shown as mean ± SD (*n* = 3-4 animals). Significance was evaluated at *p* < 0.05. *Values are significantly different from young.

Integrins α and β associate to form functional integrin dimers that bind preferentially to collagen I and fibronectin. Polymerase chain reaction was employed to determine whether aging alters gene expression for a subset of specific integrins α2, α5, αv, β1, β3 and SMα-actin in VSM cells isolated from young and old SFA. Figure 2 shows that mRNA expression for integrin β1 was significantly downregulated, while integrins α5, αv, β1, and β3 trended to be lower in old arteries relative to young arteries. Integrin α2 mRNA expression did not change with age. In addition, SMα-actin mRNA expression was also lower in old arteries.

**FIGURE 3:**
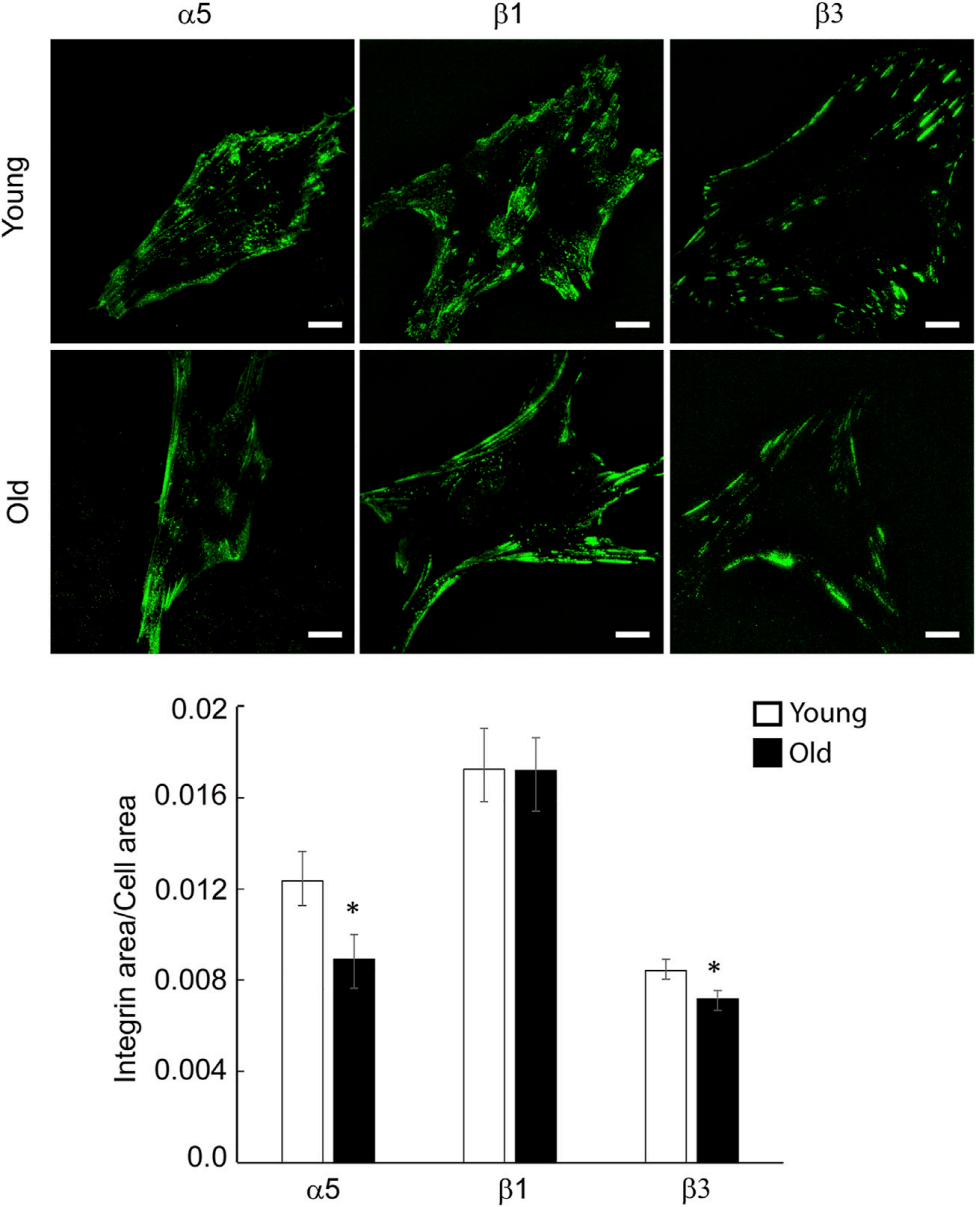
Representative TIRF images of VSM cells isolated from young and old Fischer 344 rats fluorescently labeled for integrin α5, β1 and β3 are shown. Scale bar represents 10 μm. Quantitative measurements (*n* = 29–39) are presented as mean ± SE. *Significance was evaluated at *p* < 0.05.

To determine the effect of aging on specific integrin localization at cell-matrix adhesions, we performed total internal reflection fluorescence (TIRF) imaging of young and old VSM cells plated on uncoated substrates and stained with specific integrin antibodies for a subset of these integrin subunits α5, β1 and β3 (Figure 3). While both young and old cells present streak-like adhesions around cell edges, integrin α5 and β1 are also present to a lesser extent towards basal cell interior for old cells. Quantitative image analysis shows that integrin α5 and β3 recruitment at cell-matrix adhesions decreases significantly and moderately, respectively, in old cells, with no change in integrin β1 with age. Taken together, these data suggest that aging decreases integrin gene expression and elicits differential protein recruitment at cell-matrix adhesions.

#### 3.3.2 Matrix Modulates Specific Integrin Recruitment at Cell-Matrix Adhesions

**FIGURE 4:**
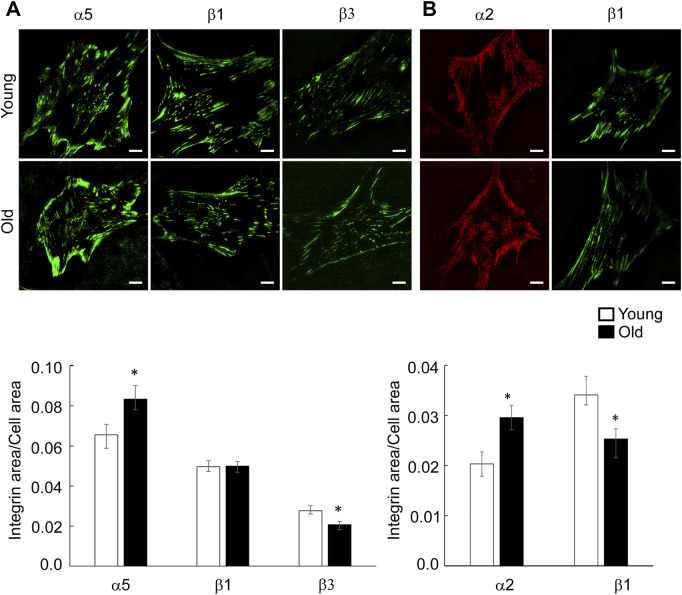
**(A)** Representative TIRF images of VSM cells plated on fibronectin and fluorescently labeled for integrin α5, β1 and β3 are shown. **(B)** Representative TIRF images of VSM cells plated on collagen-I and fluorescently labeled for integrin α2 (red) and β1 (green) are shown. Scale bar represents 10 μm. Quantitative measurements (*n* = 15–50) are presented as mean ± SE. Significance was evaluated at *p* < 0.05.

VSM cells predominantly express fibronectin binding integrins α5β1, αvβ3 and α4β1 and collagen binding integrins α1β1 and α2β1, both matrix components being increased in the aged vessel wall (Qiu et al., 2010; Lakatta 2013; Sehgel et al., 2015a). To determine the matrix effect on specific integrin distribution at cell-matrix adhesions in aging, we performed TIRF imaging of young and old VSM cells plated on fibronectin and collagen I and stained with specific integrin antibodies for integrin α2, α5, β1 and β3. As shown in Figure 4, integrin α5 and β1 are present both at cell edges, forming streak-like adhesions, and towards the cell center for cells plated on FN, an area known for the association of integrin α5β1 with fibronectin fibrilogenesis (Zhong et al., 1998; Pankov et al., 2000). However, recruitment of integrin α5 at cell edges is higher in old cells, with reduced presence towards the cell interior where it forms smaller, dot-like adhesions. For cells plated on collagen I, integrin α2 and β1 localize mostly at cell edges. Quantitative analysis of TIRF images showed that for cells plated on fibronectin, integrin α5 recruitment at cell adhesions is higher in old cells, while integrin β3 is lower with no change for integrin β1 (Figure 4A). For cells plated on collagen I, integrin α2 presents higher recruitment in older cells, while integrin β1 is significantly lower (Figure 4B). Since individual integrins pair to form functional dimers, these data suggest that matrix-dependent age-induced integrin recruitment increases integrin α5β1 and α2β1 at cell-matrix adhesions when cells are plated on exogenous fibronectin and collagen functionalized substates, respectively, while integrin αvβ3 is decreased in cells plated on fibronectin.

#### 3.3.3 Aging Modulates Functional Integrin Binding to the Matrix

**FIGURE 5:**
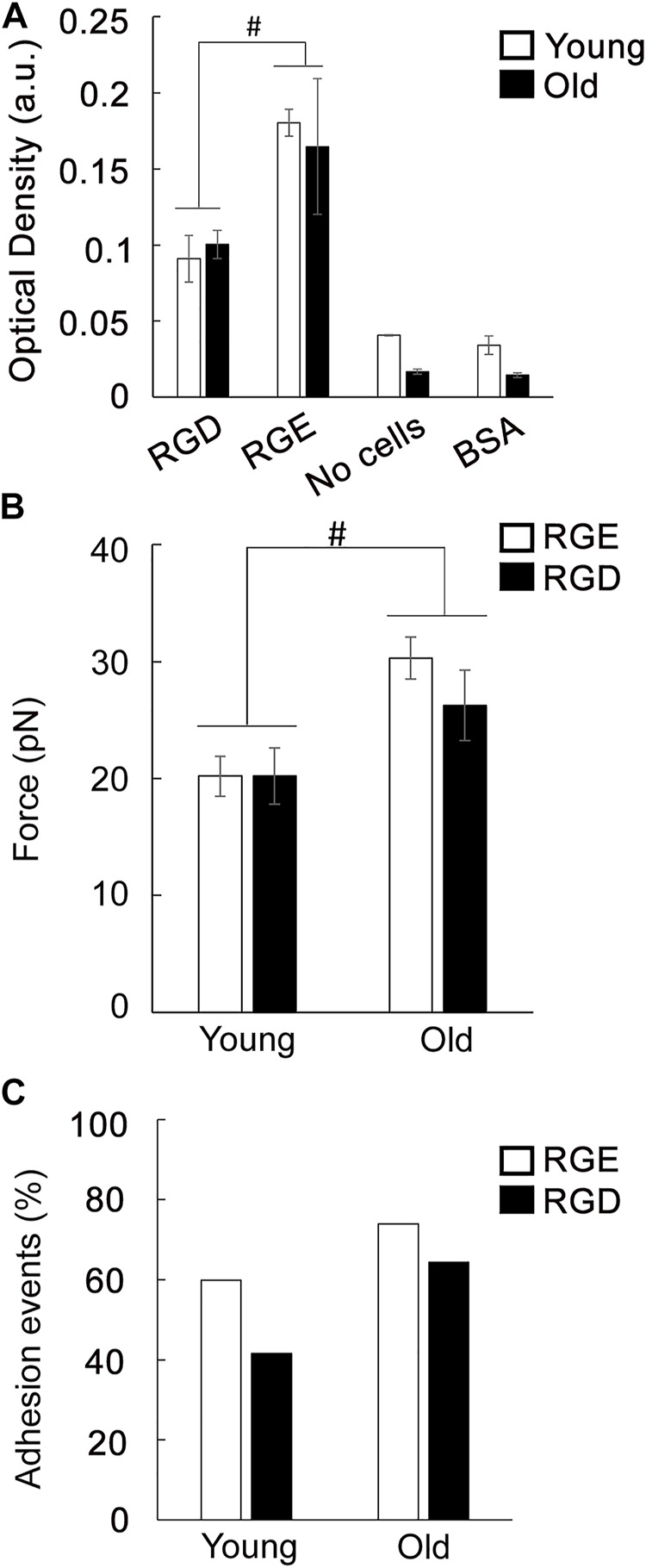
**(A)** Adhesion assay of VSM cells to fibronectin was performed in the presence of a functional integrin blocking RGD peptide and a non-blocking RGE peptide. Negative control experiments were performed using untreated cells plated on substrates blocked with BSA, and specific matrix without cells. Data are presented as mean ± SEM. **(B)** Functional AFM measurements of integrin α5β1 binding force to fibronectin for young and old VSM cells treated with a functional integrin blocking RGD peptide and a non-blocking RGE peptide are shown. For comparisons, force peak values whose confidence intervals did not overlap were considered significantly different (*p* < 0.05). **(C)** Percent of adhesion events for each treatment is shown.

To further investigate if aging affects the functional role of integrin receptors involved in VSM cell adhesion to the matrix, cell adhesion assays were performed using RGD inhibitory peptide and RGE as control peptide (Figure 5A). Treatment of VSM cells in suspension with RGD reduced cell adhesion to fibronectin 2-fold in both young and old cells in respect with RGE non-inhibitory peptide. In addition, AFM experiments performed with fibronectin functionalized probes investigated single ligand-receptor interaction in the presence of each of the peptides. These discrete measurements showed that dhesion strength of α5β1 integrin to fibronectin is age-dependent with a significant ∼30% increase in old cells in respect with young ones (Figure 5B). RGD peptide added to the cell medium before performing AFM measurements will bind to the integrins on the cell surface and reduce the number of integrins able to further bind to the fibronectin functionalized AFM probe. This RGD inhibition, however, will not affect adhesion binding force of the remaining integrins able to bind the AFM probe (Trache et al., 2005).

Previously, we have shown that adhesion probability of integrin α5β1 binding to fibronectin is 20% higher in untreated old cells indicating that there are more unbound free functional integrins present on the cell surface (Seawright et al., 2018). While similar results were obtained for RGE control peptide, RGD inhibition is less effective by inducing a reduced integrin binding inhibition in old cells (15%) by comparison with young ones (30%) (Figure 5C). This differential behavior of the discrete α5β1 integrin functional inhibition in old cells may be due to an age-induced dysregulation of single ligand-receptor interaction of free integrins available to bind on the cell surface that decreases integrin α5β1 binding to soluble RGD (Lu et al., 2020), effect that may be masked by the bulk cell adhesion assay. Collectively, these data show that integrins are important receptors in regulating cell adhesion in aged cells, and aging modulates integrin function.

### 3.4 Substrate Stiffness Modulation of VSM Cells Architecture is Age-Dependent

#### 3.4.1 Integrin Expression and Spatial Distribution

Taking in consideration the reduced integrin-mediated vascular contractility with age, we asked if extracellular matrix or its stiffness may affect integrin expression and recruitment at cell-matrix adhesions in VSM cells.

**FIGURE 6:**
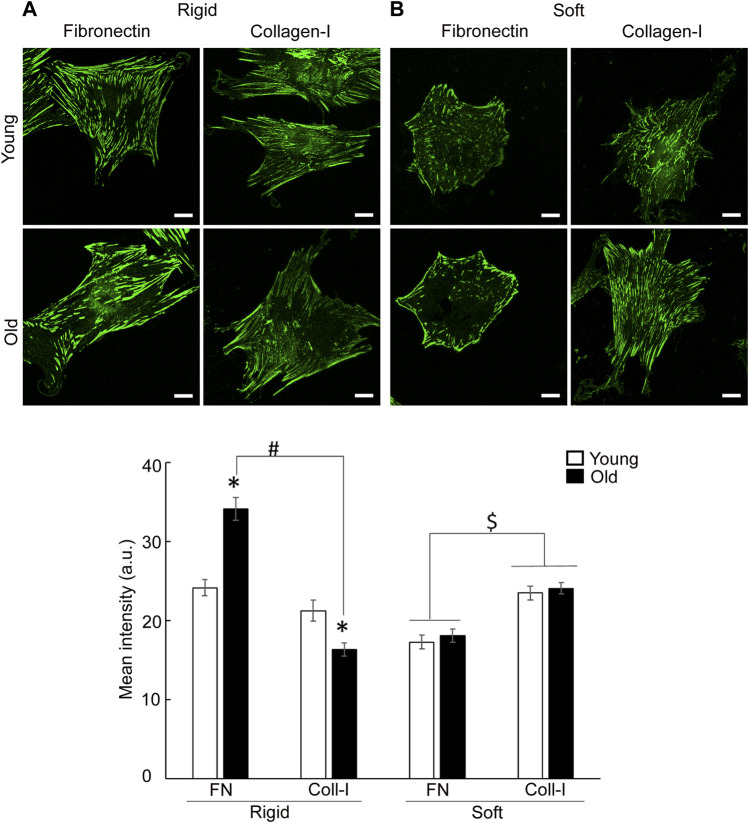
Representative confocal images of VSM cells plated on **(A)** rigid and **(B)** soft substrates functionalized with fibronectin and collagen-I, respectively, are shown. VSM cells have been fluorescently labeled for integrin β1. Scale bar represents 10 μm. Quantitative measurements of overall fluorescence intensity (*n* = 28–68) are presented as mean ± SE. Significance was evaluated at *p* < 0.05. *Values are significantly different from young. # Values are significantly different from age matched VSM cells on different matrices. $ Values are significantly different between matrices.

To address the effect of matrix-functionalized substrate stiffness on integrin recruitment at cell-matrix adhesions, cells were plated on glass-bottom cell culture dishes or soft membranes (212.05 ± 0.64 kPa) functionalized with matrix proteins, fibronectin or collagen I. Since β1 integrin dimerizes with α5 to bind fibronectin and with α2 for collagen-I binding, confocal imaging was performed on VSM cells labeled with integrin β1 antibodies (Figure 6). Quantification of confocal images revealed that aging modulates overall integrin expression in a matrix-dependent manner enhancing integrin β1 (i.e., α5β1) expression for cells plated on rigid substrates functionalized with fibronectin, but reduces overall presence of integrin β1 (i.e., α2β1) in old cells plated on collagen I. However, general morphology of integrin β1 on rigid substrates follow the same characteristics of TIRF imaging presented above. Interestingly, total β1 integrin content (i.e., integrin present at cell adhesions and throughout the cytoplasm) is the same in young and old cells plated on soft substrates for either matrix, but its expression is increased for cells plated on collagen-I by comparison with fibronectin. Thus, there is a significant interplay between age effects and substrate stiffness for integrin β1 in VSM cells plated on either matrix, fibronectin or collagen-I (Supplementary Figure S1).

**FIGURE 7:**
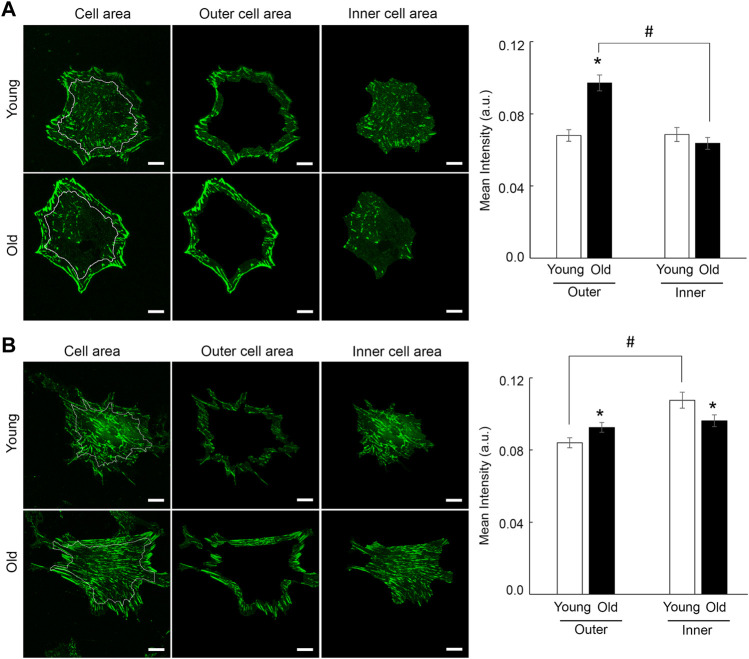
Representative confocal images of VSM cells plated on soft substrates functionalized with fibronectin **(A)** and collagen-I **(B)** are shown. VSM cells have been fluorescently labeled for integrin β1. Scale bar represents 10 μm. Quantification of fluorescence intensity relative to inner vs. outer cell area shows a strong recruitment of integrin β1 at cell edges for old cells plated on fibronectin (*n* = 28–30), with a modest relative change for cells plated on collagen-I (*n* = 40–50). Data are presented as mean ± SE. Significance was evaluated at *p* < 0.05. *Values are significantly different from young. #Values are significantly different from age matched VSM cells.

Moreover, integrin β1 presents a matrix-dependent and age-dependent spatial organization. Thus, old cells plated on soft membranes functionalized with fibronectin show strong integrin β1 localization at cell edges, while young cells distribute integrin β1 almost evenly across cell area. A masking technique that allows fluorescence quantification at cell edges (i.e., outer cell area) vs. inner cell area, confirmed the increase in β1 integrin recruitment at cell edges of old cells plated on fibronectin (Figure 7A). However, when cells are plated on membranes functionalized with collagen I, adhesions formed by integrin β1 appear as thin streak-like adhesions all over basal cell area. Quantification of fluorescence showed that old cells plated on soft membranes functionalized with collagen-I are characterized by a modest increase of integrin β1 at cell edges with respect to young cells, while inner area of young cells presents a slight increase in integrin β1 recruitment with respect to old cells (Figure 7B). Even though the shift in integrin β1 distribution is significantly different (*p* < 0.05), the overall change is less pronounced than for fibronectin. These data suggest that changes in substrate stiffness, induce a change in cell adhesion and contractile properties even if cells behave normally (i.e., young cells). Thus, substrate stiffening drives altered integrin β1 expression in aging, while soft substrates abolish age-induced differences in overall integrin β1 expression.

Collectively, these results suggest that both substrate stiffness and extracellular matrix differentially modulate specific integrin expression. Taking in consideration that α2 and α5 subunit dimerizes with β1 subunit to form functional integrin dimers, the availability of α-integrins may limit functional dimer formation, or competitive binding of β1 integrin with other α-subunits may limit β1 availability for binding.

#### 3.4.2 Actin Cytoskeleton Architecture

**FIGURE 8:**
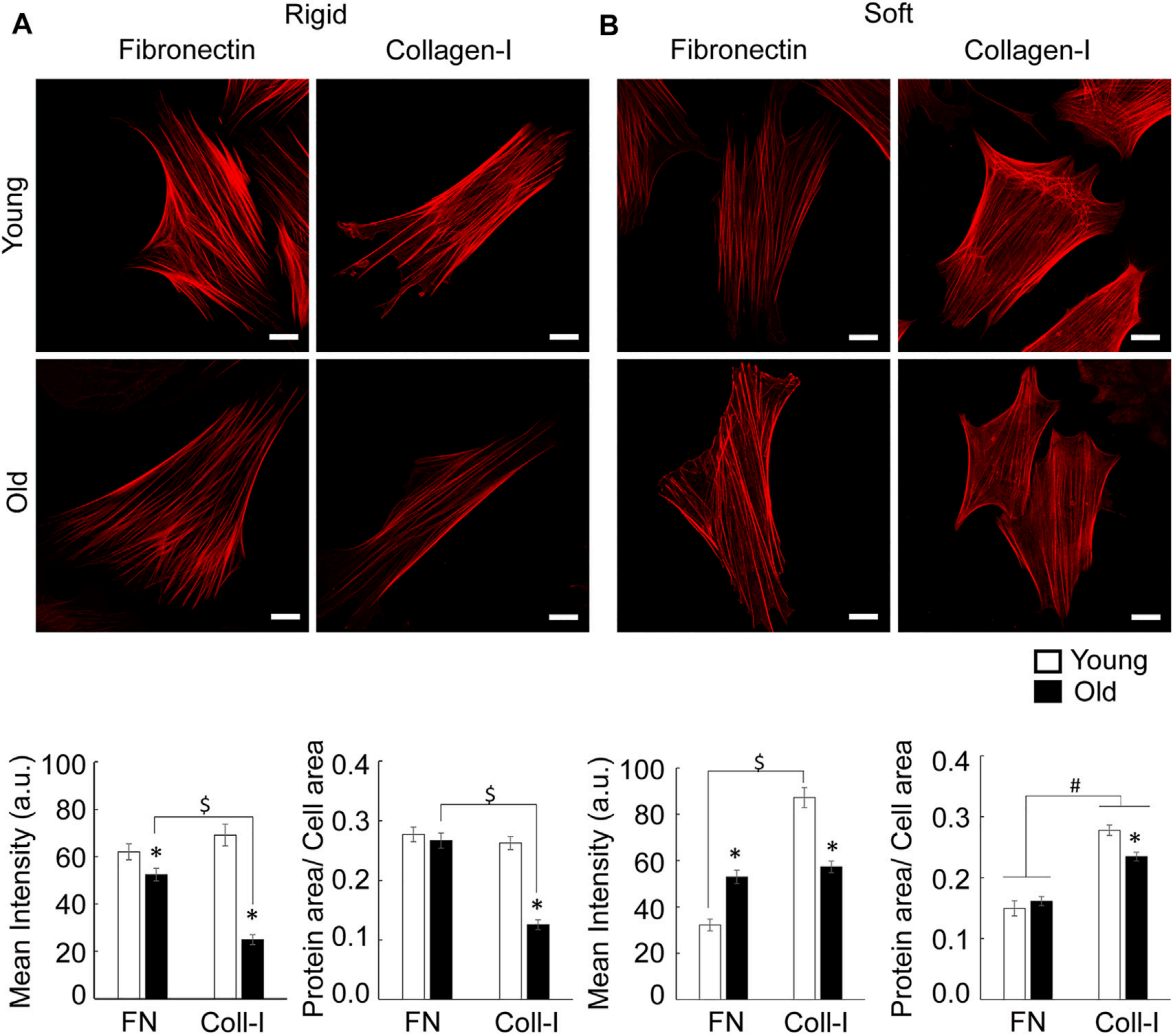
**(A)** Representative confocal images of VSM cells plated on rigid substrates functionalized with fibronectin or collagen-I are shown. VSM cells have been fluorescently labeled for SMα-actin. Scale bar represents 10 μm. Quantitative measurements of overall fluorescence intensity and actin fiber segmentation (*n* = 34–68) are presented as mean ± SE. Significance was evaluated at *p* < 0.05. **(B)** Representative confocal images of VSM cells plated on soft substrates functionalized with fibronectin or collagen-I are shown. VSM cells have been fluorescently labeled for SMα-actin. Scale bar represents 10 μm. Quantitative measurements of overall fluorescence intensity and actin fiber segmentation (*n* = 22–42) are presented as mean ± SE. Significance was evaluated at *p* < 0.05. *Values are significantly different from young. $ Values are significantly different from age matched VSM cells on different matrices. # Values are significantly different between matrices.

Since cytoskeletal architecture is directly linked to formation of cell-matrix adhesions, we further asked how substrate stiffness and extracellular matrix regulate actin stress fiber formation in aging. Consistent with previous findings (Seawright et al., 2018), young cells on rigid substrates present prominent actin fibers on either matrix, while old cells show finer fibers mostly for cells plated on collagen I (Figure 8A). This finding is consistent with higher cytoskeletal tension induced by fibronectin engagement of integrin α5β1 at cell-matrix adhesions. Quantitative analysis showed that matrix-dependent assembly of stress fibers decreased significantly for old cells plated on collagen-I. In contrast, soft membranes induced finer fibers distributed all over cell area for cells plated on fibronectin, with an additional increase in intracellular actin haze, presumably monomeric G-actin, and very fine fibers for cells plated on collagen I (Figure 8B). In addition, collagen-I induces formation of thick actin bundles at cell edges, mostly for young cells plated on soft substrates.

Quantitative analysis showed that on soft substrates, matrix has no effect on overall SMα-actin mean intensity of old cells. However, fibronectin induces a significant increase of SMα-actin mean intensity in old cells by comparison with young ones, while collagen-I has an opposite effect. In addition, soft substrates reduce age differences regarding SMα-actin stress fiber formation for cells plated on collagen-I inducing an increase in SMα-actin fibers in respect to fibronectin.

Taken together, these data suggest that substrates stiffness and matrix composition contribute to the modulation of SMα-actin cytoskeleton architecture with soft substrates reducing age-dependent actin stress fiber formation.

## 4 Discussion

Aging results in progressive changes in the mechanical properties of the arterial wall leading to increased wall stiffness and decreased responsiveness of vascular cells to mechanical stimuli. The mechanism responsible for the age-related decrease in vasoconstrictor function of resistance arteries has not been fully elucidated. The discrete VSM cell mechanical properties, cell adhesion to the matrix and their ability to adapt to external mechanical signals, directly contribute to maintaining vessel tone (Martinez-Lemus et al., 2005a; Seawright et al., 2016). However, the impaired ability of aged VSM cells to develop contractile tension, may be in part due to age-induced alterations of integrin function because of the increase in the stiffness of the extracellular matrix substrate. Our previous studies demonstrated that vasoconstrictor responsiveness declines with age in SFA (Seawright et al., 2016), and aged VSM cells were stiffer and not able to generate the force needed to induce matrix remodeling (Seawright et al., 2018). Therefore, in the current study we evaluated aging effects on integrin function and spatial distribution in the context of cell-matrix interactions. Our results showed that: 1) age-induced decrease in SFA contractile function was mediated, in part, by integrin signaling; 2) aging alters integrin recruitment and binding to the matrix; and 3) soft substrates reduce, in part, age effects on integrin expression and actin stress fibers formation.

Results from functional experiments performed on SFA showed that constrictor responses to NE, PE and Ang II were significantly impaired in old SFA relative to young SFA (Figure 1). Results also revealed that RGD had a significant inhibitory effect on constrictor responses in young and old SFA, indicating that integrin signaling contributes to the vasoconstrictor responses. The inhibitory effect of the RGD peptide varied with the agonist. Specifically, RGD inhibition was lowest for PE-induced constriction and highest for Ang II-induced constriction. The differences in the RGD inhibition levels likely reflected engagement of different receptor types. PE was used to selectively activate alpha-1 adrenergic receptors whereas NE was used to activate alpha-1 and alpha-2 adrenergic receptors. By comparison, Ang II activated angiotensin (AT) receptors. Our finding that NE and PE-induced constriction was attenuated (not abolished) in the presence of RGD indicates that integrin signaling contributed to, but did not fully account for, adrenergic receptor-mediated constrictor responses. However, our results showed that the RGD reduced constrictor responses to Ang II in young (78%) and old (92%) SFA, suggesting that the RGD nearly abolished constrictor responses to Ang II in old SFA. This indicates that angiotensin receptor-mediated constriction in old SFA was mostly dependent on integrin signaling. Since Ang II levels are increased with aging (Yoon et al., 2016), integrin activation *via* AT1 receptor plays an important role in regulating SFA contractility and stiffness in old vessels (De Luca 2019). This observation is in good agreement with other studies showing a positive correlation between Ang II effects via AT1 receptor and integrin activation in different vascular beds (Kawano et al., 2000; Jia et al., 2003; Brassard et al., 2006; Bunni et al., 2011).

Aging of the vessel wall correlates with changes in VSM cell mechanics and architecture. Changes in mechanical properties of aged VSM cells may induce physiological deficiencies resulting in a reduced ability of the cells to sense and transduce mechanical cues into intracellular biochemical signals necessary to maintain cellular homeostasis (Phillip et al., 2015). Key regulators of the VSM cell architecture are the cell-matrix adhesions and actin cytoskeleton. Our previous studies showed that aging decreases recruitment of key adhesion proteins, vinculin and pFAK, at cell-matrix adhesions and formation of SMα-actin stress fibers in VSM cells (Seawright et al., 2018). However, at the same time, an increase in strength of cell adhesion to the matrix in aged VSM cells may be an important contributing factor to arterial stiffening in aging (Qiu et al., 2010; Sehgel et al., 2015b). Since integrins are the main mechanotransducers connecting the actin cytoskeleton to the matrix, we assessed how aging modulates integrin recruitment and function at cell-matrix adhesions. Molecular studies revealed a general trend that aging downregulates a subset of α- and β-integrins and SMα-actin gene expression, with significant downregulation for integrin β1 gene expression (Figure 2). While this downregulation was confirmed by reduced recruitment of integrin α5 and β3 at cell adhesions in VSM cells in control conditions, integrin β1 showed no changes with age (Figure 3).

Since both fibronectin and collagen I are upregulated in aging, we asked if integrin function may be altered by age-induced changes in extracellular matrix composition. Plating of VSM cells on exogenous fibronectin enhanced the presence of integrin α5 toward cell interior in young cells showing an active fibrilogenesis process (Zhong et al., 1998; Pankov et al., 2000). In contrast, in old cells plated on fibronectin, integrin α5 is enhanced at cell edges, while integrin β3 presence at cell edges is decreased. This age-induced alteration of integrin spatial distribution at cell-matrix adhesions may be associated with abnormal age-induced maturation of cell-matrix adhesions (Figure 4), that could eventually increase fixation of the cell within the surrounding tissue and reduce its ability to adapt to mechanical cues. These data are supported by our previous observation that aged cells present an increased α5β1 integrin adhesion to fibronectin, but a decrease in FAK-p397 activation at peripheral cell-matrix adhesions (Seawright et al., 2018). Since VSM cells respond to extracellular mechanical cues with each contraction-relaxation cycle to ensure proper vessel wall function (Martinez-Lemus et al., 2009) an increased cell adhesion and a decrease in FAK phosphorylation at cell edges may be important factors in reducing vessel wall contractility in aged arteries. In addition, integrin α5β1 has an important role in fibronectin fibrillogenesis, and cells exert force on fibronectin molecules to organize fibronectin fibers (Zhong et al., 1998; Pankov et al., 2000). Our results show that even though RGD inhibitory peptide effectively inhibited young and old cells binding to fibronectin matrix (Figure 5A), aging dysregulates the ability of discrete integrin α5β1 binding to fibronectin (Figure 5B) that may indicate reduced fibrilogenesis in old cells and a switch to a synthetic phenotype. Furthermore, VSM cells plating on exogenous collagen-I matrix elicited expression of α2β1 integrin necessary for binding and proper VSM cell anchorage to the matrix. However, aged cells presented less β1 integrin at cell-matrix adhesions despite an enhanced expression of α2 integrin. Since integrin β1 subunit has also other partners, this imbalance of β1 integrin availability may induce a weak binding of VSM cell to the collagen-I matrix. These results are in agreement with our previous 3D contractility assays in which aged VSM cells embedded in collagen-I fibrous hydrogels failed to remodel the matrix, hence, a weak attachment to the matrix will impede generation of the needed force on the matrix (Seawright et al., 2018).

Cells adhering to the extracellular matrix not only engage specific integrins but also probe their immediate mechanical microenvironment by sensing the substrate mechanical properties and adapting its cytoskeletal tension to maintain cellular homeostasis. Mechanical properties of the substrate provide a physical cue that induces an outside-in mechanical signaling to integrins, and thus, regulates cell-matrix adhesion assembly and intracellular signaling. In tissues, changes in substrate mechanical properties are characterized by changes of the stiffness of the extracellular matrix. Quantitative confocal imaging analysis performed on young and old cells plated on soft and rigid substrates functionalized with fibronectin or collagen, showed that changes in substrate stiffness induced a change in both cell adhesion and actin stress fiber formation. Specific β1 integrin staining revealed both matrix- and age-dependent spatial organization. In contrast with rigid substrates, the soft substrates elicited the same integrin β1 expression for both young and old cells on either matrix, with a higher expression of integrin β1 for cells plated on collagen-I by comparison with fibronectin (Figure 6). However, in comparison with young cells, integrin β1 recruitment is prominent at cell edges for old cells plated on fibronectin, while a moderate recruitment at cell periphery was found for collagen-I (Figure 7). We suggest that presentation of old cells with a soft substrate recovers integrin expression in aging and partly restores integrin-dependent intracellular signaling. Indeed, quantification of SMα-actin fibers for old cells plated on soft substrates, show a similar trend with integrin β1 expression in the same conditions. Integrin α5β1 associates with SMα-actin stress fibers and is part of the matrix-integrin-actin axis responsible for regulating cellular contractility in VSM cells. A study by Fusco et al. (2015) showed that hydrogels of different stiffnesses functionalized with RGD peptides have been shown to regulate formation of cell-matrix adhesions in a substrate-stiffness dependent manner. Their observations that thicker stress fibers were associated with larger adhesions is in good agreement with our results. In addition, the age-induced decrease in SMα-actin fibers formation for cells plated on collagen-I functionalized rigid substrates may relate to the old cell deficient ability to properly organize actin cytoskeleton in the presence of collagen-I matrix. Since collagen-I is increased in aging, this result would be in good agreement with the reduction in contractility of the aged VSM.

In summary, the results of this study demonstrate that integrins are important receptors in modulating cell adhesion in aged VSM cells, and dysregulation of integrin function in aging is due, in part, to stiffening of the extracellular matrix. Deficient assembly of cell adhesions provides a feedback loop for the integrin-mediated signal transduction and further actin cytoskeleton remodeling (Phillip et al., 2015). These age-induced discrete changes of VSM cell structure and function lead to physiological dysfunctions at the vessel level and contribute, in part, to the reduced contractility of aged resistance arteries.

## Data Availability

The original contributions presented in the study are included in the article/Supplementary Material. Further inquiries can be directed to the corresponding author.

## Supplementary Material

Supplemental Figure 1 Box plots of quantitative measurements of overall mean fluorescence intensity measurements of integrin β1 presented in Figure 6 for VSM cells plated on **(A)** fibronectin and **(B)** collagen-I, respectively. *Significance was evaluated at *p* < 0.05.

Supplemental Table 1 RGD inhibition effect on reducing functional constrictor responses to NE, PE and Ang II in young and old SFA for the data presented in Figure 1. Estimated % RGD inhibition in respect to control and the 95% confidence intervals are shown. For comparisons, values whose confidence intervals did not overlap were considered significantly different (*p* < 0.05).

## References

[B1] AlGhatrif M. Strait J. B. Morrell C. H. Canepa M. Wright J. Elango P. (2013). Longitudinal Trajectories of Arterial Stiffness and the Role of Blood Pressure. Hypertension 62 (5), 934–941. 10.1161/hypertensionaha.113.01445 24001897 PMC3880832

[B2] Barton M. Cosentino F. Brandes R. P. Moreau P. Shaw S. Lüscher T. F. (1997). Anatomic Heterogeneity of Vascular Aging. Hypertension 30 (4), 817–824. 10.1161/01.hyp.30.4.817 9336378

[B3] Brassard P. Amiri F. Thibault G. Schiffrin E. L. (2006). Role of Angiotensin Type-1 and Angiotensin Type-2 Receptors in the Expression of Vascular Integrins in Angiotensin II-Infused Rats. Hypertension 47 (1), 122–127. 10.1161/01.hyp.0000196272.79321.11 16330679

[B4] Briones A. M. Salaices M. Vila E. (2007). Mechanisms Underlying Hypertrophic Remodeling and Increased Stiffness of Mesenteric Resistance Arteries from Aged Rats. Journals Gerontology Ser. A Biol. Sci. Med. Sci. 62 (7), 696–706. 10.1093/gerona/62.7.696 17634315

[B5] Bunni M. A. Kramarenko II Walker L. Raymond J. R. Garnovskaya M. N. (2011). Role of Integrins in Angiotensin II-Induced Proliferation of Vascular Smooth Muscle Cells. Am. J. Physiology-Cell Physiology 300 (3), C647–C656. 10.1152/ajpcell.00179.2010 PMC3063971 21148411

[B77] D’Angelo G. Mogford J. E. Davis G. E. Davis M. J. Meininger G. A. (1997). Integrin-Mediated Reduction in Vascular Smooth Muscle [Ca^2+^]i Induced by RGD-Containing Peptide. Am. J. Physiol. 272 (4 Pt 2), H2065–H2070. 10.1152/ajpheart.1997.272.4.H2065 9139994

[B7] De Luca M. (2019). The Role of the Cell-Matrix Interface in Aging and its Interaction with the Renin-Angiotensin System in the Aged Vasculature. Mech. Ageing Dev. 177, 66–73. 10.1016/j.mad.2018.04.002 29626500 PMC6170735

[B8] Discher D. E. Janmey P. Wang Y.-l. (2005). Tissue Cells Feel and Respond to the Stiffness of Their Substrate. Science 310 (5751), 1139–1143. 10.1126/science.1116995 16293750

[B9] Felsenfeld D. P. Schwartzberg P. L. Venegas A. Tse R. Sheetz M. P. (1999). Selective Regulation of Integrin-Cytoskeleton Interactions by the Tyrosine Kinase Src. Nat. Cell. Biol. 1 (4), 200–206. 10.1038/12021 10559917

[B78] Fusco S. Panzetta V. Embrione V. Netti P. A. (2015). Crosstalk Between Focal Adhesions and Material Mechanical Properties Governs Cell Mechanics and Functions. Acta Biomater. 23, 63–71. 26004223 10.1016/j.actbio.2015.05.008

[B12] Ginsberg M. H. Du X. Plow E. F. (1992). Inside-out Integrin Signalling. Curr. Opin. Cell. Biol. 4 (5), 766–771. 10.1016/0955-0674(92)90099-x 1419055

[B13] Göthberg G. Folkow B. (1983). Age-dependent Alterations in the Structurally Determined Vascular Resistance, Pre- to Postglomerular Resistance Ratio and Glomerular Filtration Capacity in Kidneys, as Studied in Aging Normotensive Rats and Spontaneously Hypertensive Rats. Acta Physiol. Scand. 117 (4), 547–555. 10.1111/j.1748-1716.1983.tb07225.x 6880810

[B14] Gupta M. Doss B. Lim C. T. Voituriez R. Ladoux B. (2016). Single Cell Rigidity Sensing: A Complex Relationship between Focal Adhesion Dynamics and Large-Scale Actin Cytoskeleton Remodeling. Cell. Adhesion Migr. 10 (5), 554–567. 10.1080/19336918.2016.1173800 PMC5079392 27050660

[B15] Gupta M. Sarangi B. R. Deschamps J. Nematbakhsh Y. Callan-Jones A. Margadant F. (2015). Adaptive Rheology and Ordering of Cell Cytoskeleton Govern Matrix Rigidity Sensing. Nat. Commun. 6, 7525. 10.1038/ncomms8525 26109233 PMC4599139

[B18] Hynes R. O. (2002). Integrins. Cell. 110 (6), 673–687. 10.1016/s0092-8674(02)00971-6 12297042

[B19] Hynes R. O. (1992). Integrins: Versatility, Modulation, and Signaling in Cell Adhesion. Cell. 69 (1), 11–25. 10.1016/0092-8674(92)90115-s 1555235

[B21] Jia N. Okamoto H. Shimizu T. Chiba S. Matsui Y. Sugawara T. (2003). A Newly Developed Angiotensin II Type 1 Receptor Antagonist, CS866, Promotes Regression of Cardiac Hypertrophy by Reducing Integrin .BETA.1 Expression. Hypertens. Res. 26 (9), 737–742. 10.1291/hypres.26.737 14620930

[B22] Kawano H. Cody R. J. Graf K. Goetze S. Kawano Y. Schnee J. (2000). Angiotensin II Enhances Integrin and α-Actinin Expression in Adult Rat Cardiac Fibroblasts. Hypertension 35 (1), 273–279. 10.1161/01.hyp.35.1.273 10642310

[B23] Kohn J. C. Chen A. Cheng S. Kowal D. R. King M. R. Reinhart-King C. A. (2016). Mechanical Heterogeneities in the Subendothelial Matrix Develop with Age and Decrease with Exercise. J. Biomechanics 49 (9), 1447–1453. 10.1016/j.jbiomech.2016.03.016 PMC4885756 27020750

[B26] Lacolley P. Regnault V. Segers P. Laurent S. (2017). Vascular Smooth Muscle Cells and Arterial Stiffening: Relevance in Development, Aging, and Disease. Physiol. Rev. 97 (4), 1555–1617. 10.1152/physrev.00003.2017 28954852

[B27] Lakatta E. G. (2013). The Reality of Aging Viewed from the Arterial Wall☆. Artres 7 (2), 73–80. 10.1016/j.artres.2013.01.003 PMC3646655 23667404

[B28] Lim S.-M. Kreipe B. A. Trzeciakowski J. Dangott L. Trache A. (2010). Extracellular Matrix Effect on RhoA Signaling Modulation in Vascular Smooth Muscle Cells. Exp. Cell. Res. 316 (17), 2833–2848. 10.1016/j.yexcr.2010.06.010 20599954

[B29] Livak K. J. Schmittgen T. D. (2001). Analysis of Relative Gene Expression Data Using Real-Time Quantitative PCR and the 2−ΔΔCT Method. Methods 25 (4), 402–408. 10.1006/meth.2001.1262 11846609

[B30] Lu J. Doyle A. D. Shinsato Y. Wang S. Bodendorfer M. A. Zheng M. (2020). Basement Membrane Regulates Fibronectin Organization Using Sliding Focal Adhesions Driven by a Contractile Winch. Dev. Cell. 52 (5), 631–646. e634. 10.1016/j.devcel.2020.01.007 32004443 PMC8335633

[B31] Martinez-Lemus L. A. Crow T. Davis M. J. Meininger G. A. (2005b). αvβ3- and α5β1-integrin Blockade Inhibits Myogenic Constriction of Skeletal Muscle Resistance Arterioles. Am. J. Physiology-Heart Circulatory Physiology 289 (1), H322–H329. 10.1152/ajpheart.00923.2003 15722407

[B32] Martinez-Lemus L. A. Hill M. A. Meininger G. A. (2009). The Plastic Nature of the Vascular Wall: a Continuum of Remodeling Events Contributing to Control of Arteriolar Diameter and Structure. Physiology 24, 45–57. 10.1152/physiol.00029.2008 19196651

[B33] Martinez-Lemus L. A. Sun Z. Trache A. Trzciakowski J. P. Meininger G. A. (2005a). Integrins and Regulation of the Microcirculation: from Arterioles to Molecular Studies Using Atomic Force Microscopy. Microcirculation 12 (1), 99–112. 10.1080/10739680590896054 15804978

[B34] Martinez-Lemus L. A. Wu X. Wilson E. Hill M. A. Davis G. E. Davis M. J. (2003). Integrins as Unique Receptors for Vascular Control. J. Vasc. Res. 40 (3), 211–233. 10.1159/000071886 12902635

[B35] McDaniel D. P. Shaw G. A. Elliott J. T. Bhadriraju K. Meuse C. Chung K.-H. (2007). The Stiffness of Collagen Fibrils Influences Vascular Smooth Muscle Cell Phenotype. Biophysical J. 92 (5), 1759–1769. 10.1529/biophysj.106.089003 PMC1796816 17158565

[B36] Mitchell G. F. Parise H. Benjamin E. J. Larson M. G. Keyes M. J. Vita J. A. (2004). Changes in Arterial Stiffness and Wave Reflection with Advancing Age in Healthy Men and Women. Hypertension 43 (6), 1239–1245. 10.1161/01.hyp.0000128420.01881.aa 15123572

[B37] Moiseeva E. (2001). Adhesion Receptors of Vascular Smooth Muscle Cells and Their Functions. Cardiovasc Res. 52 (3), 372–386. 10.1016/s0008-6363(01)00399-6 11738054

[B38] Na S. Trache A. Trzeciakowski J. Sun Z. Meininger G. A. Humphrey J. D. (2008). Time-dependent Changes in Smooth Muscle Cell Stiffness and Focal Adhesion Area in Response to Cyclic Equibiaxial Stretch. Ann. Biomed. Eng. 36 (3), 369–380. 10.1007/s10439-008-9438-7 18214679

[B39] Najjar S. S. Scuteri A. Lakatta E. G. (2005). Arterial Aging. Hypertension 46 (3), 454–462. 10.1161/01.hyp.0000177474.06749.98 16103272

[B40] O'Rourke M. F. Safar M. E. (2005). Relationship between Aortic Stiffening and Microvascular Disease in Brain and Kidney: Cause and Logic of Therapy. Hypertension 46 (1), 200–204. 10.1161/01.HYP.0000168052.00426.65 15911742

[B43] Pankov R. Cukierman E. Katz B.-Z. Matsumoto K. Lin D. C. Lin S. (2000). Integrin Dynamics and Matrix Assembly. J. Cell. Biol. 148 (5), 1075–1090. 10.1083/jcb.148.5.1075 10704455 PMC2174533

[B45] Phillip J. M. Aifuwa I. Walston J. Wirtz D. (2015). The Mechanobiology of Aging. Annu. Rev. Biomed. Eng. 17 (1), 113–141. 10.1146/annurev-bioeng-071114-040829 26643020 PMC4886230

[B46] Qiu H. Zhu Y. Sun Z. Trzeciakowski J. P. Gansner M. Depre C. (2010). Short Communication: Vascular Smooth Muscle Cell Stiffness as a Mechanism for Increased Aortic Stiffness with Aging. Circ. Res. 107 (5), 615–619. 10.1161/circresaha.110.221846 20634486 PMC2936100

[B48] Ripley B. D. Venables W. N. (1994). Modern Applied Statistics with S-Plus. New York: Springer-Verlag.

[B49] Rizzoni D. Porteri E. Guefi D. Piccoli A. Castellano M. Pasini G. (2000). Cellular Hypertrophy in Subcutaneous Small Arteries of Patients with Renovascular Hypertension. Hypertension 35 (4), 931–935. 10.1161/01.hyp.35.4.931 10775564

[B50] Ruoslahti E. (1996). RGD and Other Recognition Sequences for Integrins. Annu. Rev. Cell. Dev. Biol. 12 (1), 697–715. 10.1146/annurev.cellbio.12.1.697 8970741

[B72] Saphirstein R. J. Gao Y. Z. Jensen M. H. Gallant C. M. Vetterkind S. Moore J. R. (2013). The Focal Adhesion: A Regulated Component of Aortic Stiffness. PLoS One 8 (4), e62461. 23626821 10.1371/journal.pone.0062461 PMC3633884

[B74] Schiller H. B. Hermann M.-R. Polleux J. Vignaud T. Zanivan S. Friedel C. C. (2013). β1- and αv-Class Integrins Cooperate to Regulate Myosin II during Rigidity Sensing of Fibronectin-Based Microenvironments. Nat. Cell Biol. 15 (6), 625–636. 23708002 10.1038/ncb2747

[B51] Seawright J. W. Sreenivasappa H. Gibbs H. C. Padgham S. Shin S. Y. Chaponnier C. (2018). Vascular Smooth Muscle Contractile Function Declines with Age in Skeletal Muscle Feed Arteries. Front. Physiol. 9, 856. 10.3389/fphys.2018.00856 30108507 PMC6079263

[B52] Seawright J. W. Trache A. Wilson E. Woodman C. R. (2016). Short-duration Increases in Intraluminal Pressure Improve Vasoconstrictor Responses in Aged Skeletal Muscle Feed Arteries. Eur. J. Appl. Physiol. 116 (5), 931–937. 10.1007/s00421-016-3350-x 26976132

[B73] Seetharaman S. Etienne-Manneville S. (2018). Integrin Diversity Brings Specificity in Mechanotransduction. Biol. Cell 110 (3), 49–64. 29388220 10.1111/boc.201700060

[B53] Sehgel N. L. Sun Z. Hong Z. Hunter W. C. Hill M. A. Vatner D. E. (2015b). Augmented Vascular Smooth Muscle Cell Stiffness and Adhesion when Hypertension Is Superimposed on Aging. Hypertension 65 (2), 370–377. 10.1161/hypertensionaha.114.04456 25452471 PMC4289111

[B54] Sehgel N. L. Vatner S. F. Meininger G. A. (2015a). "Smooth Muscle Cell Stiffness Syndrome"-Revisiting the Structural Basis of Arterial Stiffness. Front. Physiol. 6, 335. 10.3389/fphys.2015.00335 26635621 PMC4649054

[B58] Sun Z. Martinez-Lemus L. A. Trache A. Trzeciakowski J. P. Davis G. E. Pohl U. (2005). Mechanical Properties of the Interaction between Fibronectin and α5β1-integrin on Vascular Smooth Muscle Cells Studied Using Atomic Force Microscopy. Am. J. Physiology-Heart Circulatory Physiology 289 (6), H2526–H2535. 10.1152/ajpheart.00658.2004 16100245

[B59] Tomiyama H. Arai T. Koji Y. Yambe M. Motobe K. Zaydun G. (2004). The Age-Related Increase in Arterial Stiffness Is Augmented in Phases According to the Severity of Hypertension. Hypertens. Res. 27 (7), 465–470. 10.1291/hypres.27.465 15302982

[B60] Trache A. Lim S.-M. (2009). Integrated Microscopy for Real-Time Imaging of Mechanotransduction Studies in Live Cells. J. Biomed. Opt. 14 (3), 034024. 10.1117/1.3155517 19566317

[B61] Trache A. Massett M. P. Woodman C. R. (2020). Vascular Smooth Muscle Stiffness and its Role in Aging. Curr. Top. Membr. 86, 217–253. 10.1016/bs.ctm.2020.08.008 33837694

[B62] Trache A. Meininger G. A. (2008). Atomic Force Microscopy (AFM). Curr. Protoc. Microbiol. Chapter 2, Unit 2C.2. 10.1002/9780471729259.mc02c02s8 18770536

[B63] Trache A. Trzeciakowski J. P. Gardiner L. Sun Z. Muthuchamy M. Guo M. (2005). Histamine Effects on Endothelial Cell Fibronectin Interaction Studied by Atomic Force Microscopy. Biophysical J. 89 (4), 2888–2898. 10.1529/biophysj.104.057026 PMC1366785 16055535

[B64] Virani S. S. Alonso A. Benjamin E. J. Bittencourt M. S. Callaway C. W. Carson A. P. (2020). American Heart Association Council on, E., Prevention Statistics, C., & Stroke Statistics, SHeart Disease and Stroke Statistics-2020 Update: A Report from the American Heart Association. Circulation 141 (9), e139–e596. 10.1161/cir.0000000000000757 31992061

[B76] Wu X. Davis G. E. Meininger G. A. Wilson E. Davis M. J. (2001). Regulation of the L-Type Calcium Channel by Alpha 5beta 1 Integrin Requires Signaling between Focal Adhesion Proteins. J. Biol. Chem. 276 (32), 30285–30292. 11382763 10.1074/jbc.M102436200

[B75] Wu X. Mogford J. E. Platts S. H. Davis G. E. Meininger G. A. Davis M. J. (1998). Modulation of Calcium Current in Arteriolar Smooth Muscle by Alphav Beta3 and Alpha5 Beta1 Integrin Ligands. J. Cell Biol. 143 (1), 241–252. 9763435 10.1083/jcb.143.1.241 PMC2132802

[B65] Yoon H. E. Kim E. N. Kim M. Y. Lim J. H. Jang I.-A. Ban T. H. (2016). Age-Associated Changes in the Vascular Renin-Angiotensin System in Mice. Oxidative Med. Cell. Longev. 2016, 6731093. 10.1155/2016/6731093 PMC4855022 27200147

[B66] Zeltz C. Gullberg D. (2016). The Integrin-Collagen Connection-Aa Glue for Tissue Repair? J. Cell. Sci. 129 (4), 653–664. 10.1242/jcs.180992 26857815

[B67] Zhong C. Chrzanowska-Wodnicka M. Brown J. Shaub A. Belkin A. M. Burridge K. (1998). Rho-mediated Contractility Exposes a Cryptic Site in Fibronectin and Induces Fibronectin Matrix Assembly. J. Cell. Biol. 141 (2), 539–551. 10.1083/jcb.141.2.539 9548730 PMC2148448

[B71] Zhu W. Kim B. C. Wang M. Huang J. Isak A. Bexiga N. M. (2018). TGFβ1 Reinforces Arterial Aging in the Vascular Smooth Muscle Cell through a Long-Range Regulation of the Cytoskeletal Stiffness. Sci. Rep. 8 (1), 2668. 10.1038/s41598-018-20763-w 29422510 PMC5805716

