# Supplementary material for: Age-Associated Dysregulation of Integrin Function in Vascular Smooth Muscle
Source: Front Physiol. 2022 Jul 7;13:913673. doi: 10.3389/fphys.2022.913673 (PMC9301045; doi:10.3389/fphys.2022.913673)
Supplement: Supplementary file 1 [file Table1.DOCX]

|  | Age | RGD inhibition (%) | 95% confidence interval (%) |
| --- | --- | --- | --- |
| NE | Young | 52.85 | 49.54 - 56.10 |
|  | Old | 58.86 | 50.73 - 66.18 |
|  |  |  |  |
| PE | Young | 37.16 | 31.61 - 42.52 |
|  | Old | 48.31 | 35.37 - 59.16 |
|  |  |  |  |
| Ang II | Young | 78.25 | 72.46 - 83.80 |
|  | Old | 92.73 | 89.28 - 95.39 |

Supplemental Table 1
