# Supplementary figures and images for: Age-Associated Dysregulation of Integrin Function in Vascular Smooth Muscle
Source: Front Physiol. 2022 Jul 7;13:913673. doi: 10.3389/fphys.2022.913673 (PMC9301045; doi:10.3389/fphys.2022.913673)

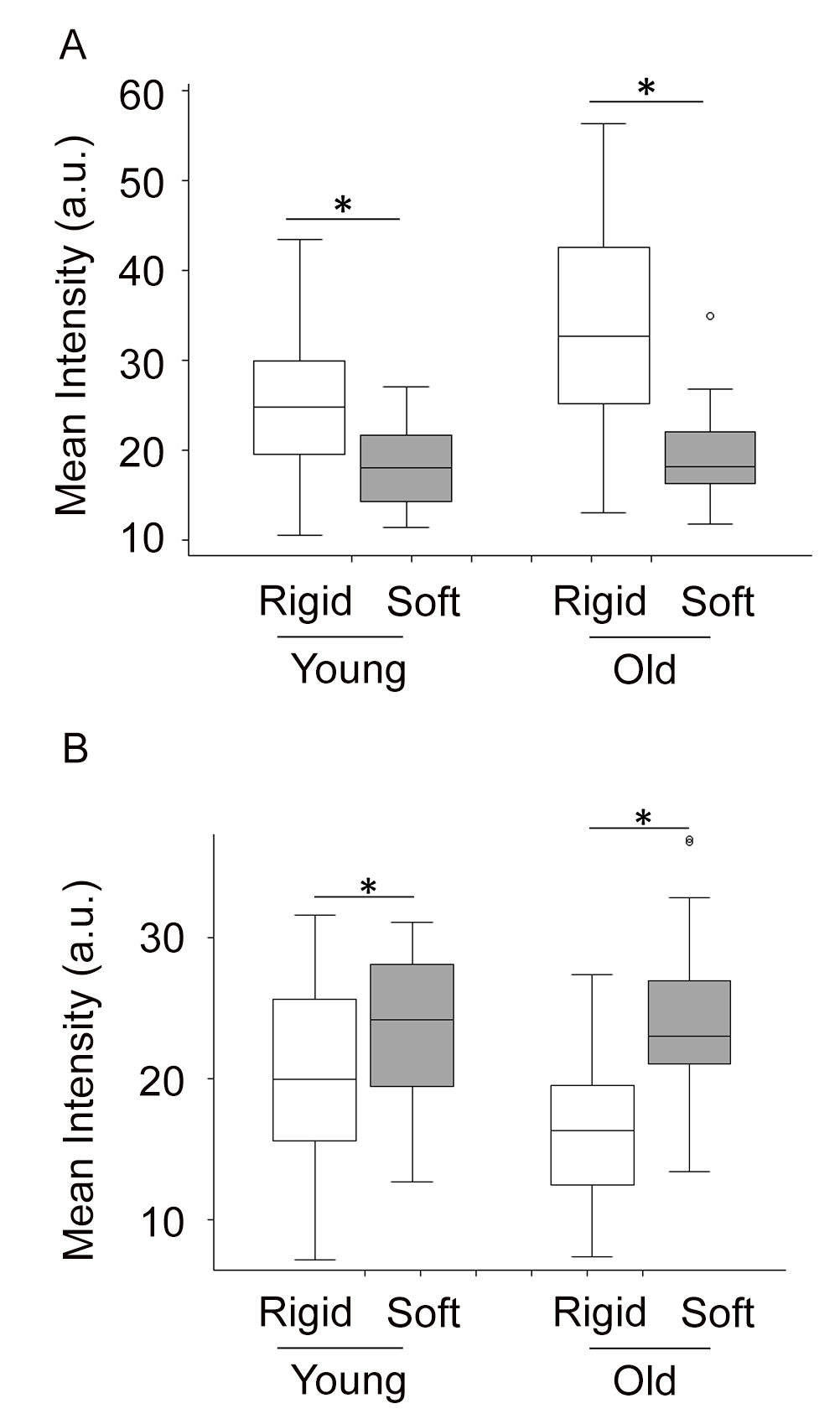

Supplement: Supplementary file 2 [file Image1.TIF]
